# Dynamic Ex Vivo Porcine Eye Model to Measure Ophthalmic Drug Penetration under Simulated Lacrimal Flow

**DOI:** 10.3390/pharmaceutics15092325

**Published:** 2023-09-15

**Authors:** Geisa N. Barbalho, Manuel A. Falcão, Jefferson M. S. Lopes, Júlia M. Lopes, Jonad L. A. Contarato, Guilherme M. Gelfuso, Marcilio Cunha-Filho, Tais Gratieri

**Affiliations:** 1 Laboratory of Food, Drugs, and Cosmetics (LTMAC), University of Brasilia, Brasília 70910-900, DF, Brazilgmgelfuso@unb.br (G.M.G.); marciliofarm@hotmail.com (M.C.-F.); 2 Physics Department, Federal University of Pará, Belém 66075-110, PA, Brazil

**Keywords:** drug penetration, ex vivo model, ocular delivery, simulated lacrimal flow

## Abstract

Animal models are still used in the research and development of ophthalmic drug products, mainly due to the difficulty in simulating natural physiological conditions with in vitro models, as there is a lack of dynamic protection mechanisms. Therefore, developing alternative ophthalmic models that evaluate drug penetration in the cornea while applying dynamic protection barriers is a contemporary challenge. This study aimed to develop a dynamic ex vivo model using porcine eyes with a simulated lacrimal flow to evaluate the performance of pharmaceutical drug products. A glass donor cell to support a simulated tear flow was designed, optimized, and custom-made. The system was challenged with different formulations (with fluconazole) including excipients with different viscosities (poloxamer 407) and mucoadhesive properties (chitosan). The results were compared to those obtained from a conventional excised cornea model mounted in Franz-type diffusion cells. The dynamic model could differentiate formulations, while the static model did not, overestimating ex vivo drug penetrated amounts. Hence, the dynamic model with simulated tear flow showed to be a simple and promising new alternative method for the drug penetration of ophthalmic formulations that ultimately can reduce the number of animals used in research.

## 1. Introduction

Animal model substitution in research, development, and routine assays of pharmaceutical drug products is a contemporary challenge [1,2]. In contrast with in vivo models, ex vivo and in vitro models can provide more reliable, faster, practical, and lower-cost results [3,4]. The development and use of these alternative techniques to in vivo animal models for the efficacy and performance evaluation of pharmaceutical drug products are being stimulated by Regulatory Health Agencies Worldwide [5].

The biggest challenge for the pharmacological efficacy of topical ophthalmic drug products is the formulation’s residence time, which is affected by the eye’s physiological characteristics and dynamic mechanisms of protection such as lacrimal flow and drainage, mucosal defence, and blinking [6,7,8,9,10,11]. However, after intimate contact of the formulation with the cornea, the second challenge is to achieve a therapeutically relevant drug ocular penetration, circumventing the barrier function posed by the corneal structure [12,13].

Innovative formulations are being developed with the addition of penetration enhancers that alter corneal epithelium integrity and enhance drug permeability aiming to boost the effectiveness of ophthalmic drug products. For instance, viscosity-enhancing polymers like poloxamer 407 are employed to reduce precorneal drug removal. The thermosensitive polymer has the advantage of the gelling process through the change in temperature, thus being able to go from the liquid state to the gelled state when it comes into contact with the tissues, thus facilitating the application of the formulation [14]. Additionally, mucoadhesive components such as chitosan are utilized to interact with the eye’s mucous layer, further enhancing drug delivery [12,13,15,16,17,18,19]. In addition, colloidal drug delivery systems have also shown enormous potential to increase drug penetration, precorneal residence time, and prolonged retention in the eye’s conjunctival region [13,20]. Thus, experimental methods to evaluate the attributes of innovative ophthalmic formulations are essential during the research and development stage to track down the most promising approaches. Two main aspects such experiments should assess are the drug’s penetration profile through ocular tissues and the formulation’s resistance to the dynamic mechanisms of ocular protection [20,21].

Current ex vivo models used for ophthalmic drug product efficacy and performance evaluation are based on the Franz diffusion cell, which consists of a donor chamber, a tissue compartment, and a receptor media compartment [4,22,23,24]. The problem is that these static models do not mimic the eye’s dynamic defence mechanisms.

When innovative formulations are tested in static ex vivo models, there are limitations in simulating natural physiological conditions, either for their lack of dynamic protection mechanisms or the anatomical differences from the human eye. Also, these models cannot detect the drug in different compartments of the eyeball, such as the cornea, conjunctiva, or aqueous humour. For such reasons, some studies proposed the use of excised whole eyes. Excised bovine eyes have been incubated in Teflon well-containing buffer [25] or in beakers with a donor chamber on top [26,27]. Also, excised porcine or human eye has been used in the inverted position so that the cornea facing down could be in contact with the test formulation filling the Teflon well [28]. An evident limitation of these models is that the cornea is exposed to a significant excess of the test formulation, resulting in swelling and hydration, which could compromise corneal integrity. Above all, such models did not include any mechanism for simulating formulation dilution and clearance. In any case, cornea exposure to very high drug concentrations over extended periods may overestimate obtained results [27,29].

Even though whole eyes can help estimate drug permeation profile, there is no alternative model to animal use to properly evaluate the formulation performance in minimally realistic conditions [30].

Alternatively, in vitro methods may be used for the determination of the mucoadhesive strength of a formulation, e.g., tensile strength test [31,32], peel strength test [33], rheological synergism [34], and interaction with mucin particles [35]. Nevertheless, all of these methods solely assess the mucoadhesion potential, and none of them integrates a key variable component: formulation resistance towards the continuous tear flow.

Thus, considering that experimental models to evaluate the efficacy and performance of innovative ophthalmic formulations are critical for the development of new approaches to ophthalmic drug delivery systems, this study aims to develop an alternative dynamic ex vivo model that uses a porcine whole eye globe with a simulated lacrimal flow to evaluate the performance of pharmaceutical drug products. The proposed method is evaluated using FLU as model drug, incorporated in formulations already tested in rabbits in vivo and in humans, so that permeation and residence performance of such formulations are already known to be different [15,16]. 

## 2. Materials and Methods

### 2.1. Material

Fluconazole (FLU), chitosan (MW = 50–190 kDa), and poloxamer 407 were purchased from Sigma-Aldrich (Steinheim, Germany). Acetonitrile and methanol of HPLC grade were purchased from J. T. Baker (Phillipsburg, NJ, USA). Buffer 4-(2-hydroxyethyl)-1piperazineethanesulfonic acid (HEPES), sodium hydroxide, and silicon grease were purchased from Dinâmica (São Paulo, Brazil). Sodium chloride was purchased from Cromoline (São Paulo, Brazil). All analyses were performed using ultra-purified water Millipore, (Illkirch-Graffenstaden, France).

The porcine eyes used in this study were collected from Via Carnes (Formosa, Brazil). The whole eye globes were extracted after slaughter and before the scalding process. It was immediately transported to the laboratory in a thermal box. The fat tissues and blood vessels were discarded, and only the eyes with the intact cornea and sclera were used, as described by [24].

### 2.2. Drug Assay

FLU (logP: 0.5, MW: 355.34 g/mol) was analysed using HPLC/UV, adapted by [16]. Briefly, FLU was extracted from cornea samples with 5 mL of an isotonic HEPES buffer solution (25 mM, pH 7.4) in an Ultra-Turrax (Model T25 Digital, IKA, Staufen, Germany) for 30 s and filtered (0.45 µm hydrophilic filters) directly into a vial. Then, the filtered samples were injected (20.0 µL) in an isocratic system using acetonitrile, methanol, and water (15:5:80) as a mobile phase with a flow rate of 0.8 mL/min. A C18 column (150 × 4.6 mm, 5 µm, Supelco Discovery BIO Wide-Pore) with the temperature settled at 40 °C was used, and the drug was detected at 210 nm. The standard curve was selective and linear (y = 30,676x + 2843.9 and R^2^ = 0.9992) for a concentration range of 0.5–10 µg/mL. The Limit of Detection and Limit of Quantification were determined to be 0.007 and 0.021 µg/mL, respectively.

### 2.3. Formulations

FLU was chosen as a model drug to compare static and dynamic permeation studies. Seven formulations of FLU 0.2% were prepared: (I) aqueous solution (SOL); (II) 14% poloxamer gel (PLX14); (III) 16% poloxamer gel (PLX16); (IV) 20% poloxamer gel (PLX20); (V) 16% poloxamer gel and chitosan 0.5% (PLX16C050); (VII) 16% poloxamer gel and chitosan 1% (PLX16C100); and (VIII) 16% poloxamer gel and chitosan 1.25% (PLX16C125). The formulations with poloxamer (14, 16, and 20% *w*/*w*) were prepared by weighing the polymer and leaving it in contact with ultra-pure water at 4 °C for 24 h. For the formulations with chitosan (0.5, 1.0, and 1.25% *w*/*w*), an aqueous solution of acetic acid 0.5% *v*/*w* was first prepared. After the complete dissolution of chitosan, poloxamer was added to the solution and left in contact at 4 °C for 24 h, as described by [15]. 

### 2.4. Design of Dynamic Model System

**Figure 1:**
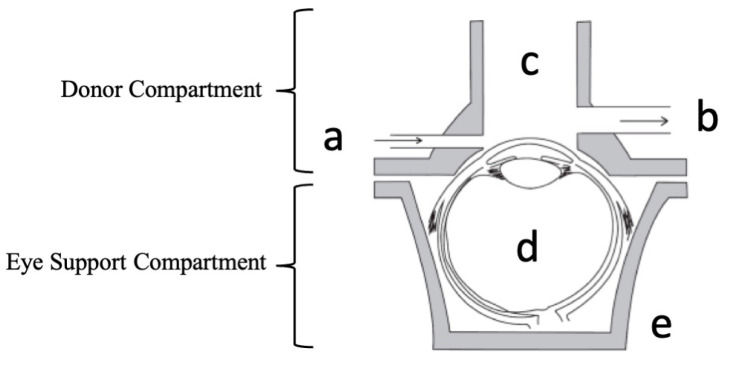
Schematic representation of the dynamic ex vivo model: the lacrimal flow channel inlet (a) and the lacrimal flow channel outlet (b), a donor compartment (c) with access to the cornea, an ex vivo porcine whole eye globe (d), and an eye support compartment (e).

To simulate the lacrimal flow in the eye, starting from a conceptual idea (Figure 1), the lacrimal flow channel inlet (a) and outlet (b), a donor compartment (c) with access to the cornea, an ex vivo porcine whole eye globe (d), and an eye support compartment (e), this system was coupled with a peristaltic pump (MINI PLUS evolution, Gilson, Middleton, WI, USA). Different glass flow donor compartments were fabricated (Unividros, Ribeirão Preto, SP, Brazil). These prototypes vary in height, angles, and width of inlet/outlet channels. To determine the maximum volume supported by the model, different volumes of dyes were applied with the aid of an automatic pipette to allow visualization of the dispersion of blue over the cornea until it reached a volume that covers the surface of the exposed cornea (300 µL). After carrying out the exploratory tests, the model was optimized considering the lacrimal flow drainage and the eyeball coupling.

Using the optimized model, ex vivo corneal penetration experiments were carried out for 15 min (2 min of 16%/min clearance + 13 min under 11%/min clearance, respectively 48 µL/min and 33 µL/min), with 300 µL (60 µg/mL) of each sample added to the corneal surface and water as simulated lacrimal flow. After the test period, the drug formulation from the corneal surface was removed with abundant water. Next, the cornea was removed from the eyeball [24] and then minced, and the drug was extracted and analysed as previously described by [16]. All experiments were carried out in quadruplicate at room temperature.

### 2.5. Static Penetration Model

Ex vivo corneal penetration experiments were performed using a Franz-type diffusion cell (diffusional area of 1.0 cm^2^). Penetration was carried out for 15 min with 300 µL of each formulation previously described, added to the corneal surface. All experiments were carried out in quintuplicate. A 15 mL volume of 25 mM HEPES buffer pH 7.4 was added to the receptor compartment. The cells were maintained at 32 ± 2 °C under magnetic stirring (500 rpm). The excised porcine cornea was positioned between the donor and receptor compartments. After the test period, the drug formulation from the corneal surface was removed with abundant water. The cornea was then minced, and the drug was extracted and analysed as previously described by [16].

### 2.6. Statistical Analyses

Data were analysed considering the analysis of variance and using GraphPad Prism^®^ Software, version 7.02 (San Diego, CA, USA). The significance level was *p* < 0.05 to reject the null hypothesis.

## 3. Results

### 3.1. Design of Dynamic Model System

**Figure 2:**
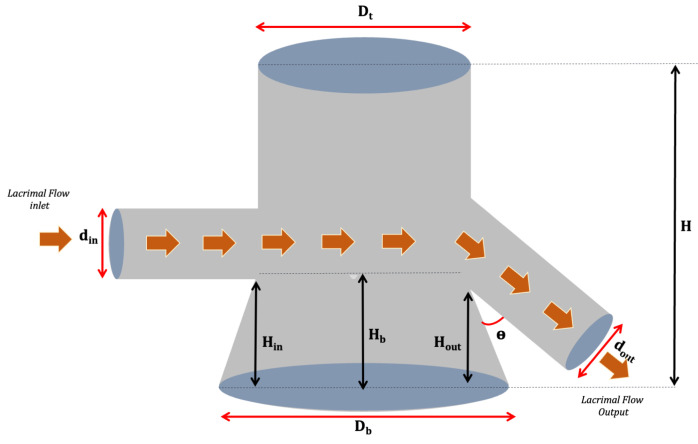
Representative schematic of the characteristic parameters of the donor medium in the dynamic model, indicating the altered dimensions. H (global height), H_in_ (inlet height), H_out_ (outlet height), H_b_ (base height), Dt (donor compartment diameter), Db (base diameter), D_out_ (outlet diameter), D_in_ (inlet diameter).

The assembly of and improvement in the dynamic model’s donor compartment included five versions. In addition, the outlet channel was produced with a larger diameter than the inlet to avoid induced pressure. As illustrated in Figure 2, the differences between the versions produced were characterized by nine parameters divided between those capable of directly affecting the simulated tear flow (associated with the horizontal axis of the donor compartment) and geometric factors related to the delivery of test formulations and accommodation of the eyeball (arranged on the vertical axis of the donor compartment). In the first case, the following are considered: (i) the mutual direction of entry and exit of the tear flow represented by the angle θ; (ii) the inlet (H_in_) and outlet (H_out_) height of the flow, and (iii) the diameter for the inlet (D_in_) and outlet (D_out_) of the flow.

In addition, regarding the geometric parameters of the donor compartment’s global height (H), two specific cases related to the internal diameter in its vertical axis were defined. In the first case, models with a single internal diameter along their entire height (D_t_ equals D_b_) were considered, characterized only by the donor compartment diameter (D_t_). In contrast, in the second case, models that have wider bases (D_b_) were used to facilitate the eyeball adjustment, leading to the final versions described by the upper diameter Dt and by a new, larger diameter defined as the base diameter (D_b_) (Figure 2, Table 1). Then, the height from which the broader donor medium (H_b_) starts was considered, as this parameter has considerably impacted the eyeball fit. Initially, the direction of tear flow was established as collinear, i.e., the inlet and outlet flow followed the same direction, forming an angle of 90° in relation to the vertical axis (height of the donor compartment).

**Figure 3:**
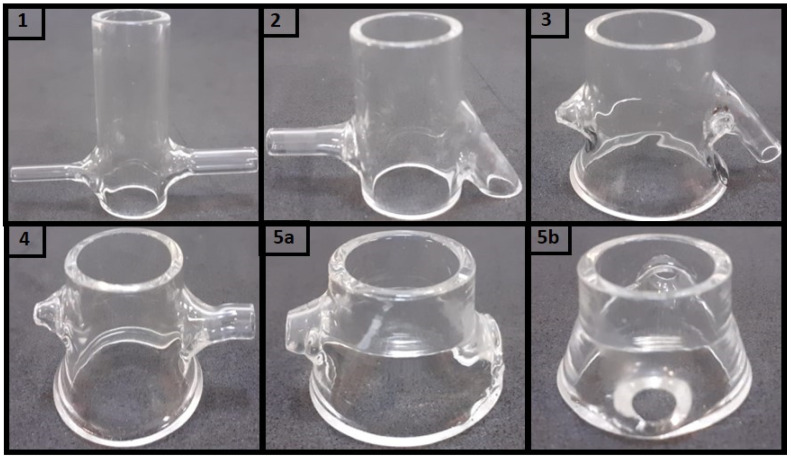
Images of the glass versions of the donor compartment: (**1**) first model without Db and angle θ = 90°; (**2**) second model without Db and angle θ < 90°; (**3**) third model with Db and angle θ < 90°; (**4**) fourth model with Db and angle θ = 90°; (**5a**) final model with Db and angle θ < 90° (side view), and (**5b**) final model with large output lacrimal flow (back view). The images are at different scales.

Two critical parameters of this model were (I) the height of the outlet, which determines the maximum volume of fluid accumulated on the ocular surface, and (II) the pump flow, which determines the drainage of the formulation (Figure 3). The design helped to reduce the amount of dammed fluid in the donor compartment. It was important to note that although versions 2 and 3 had a lower overall height of H about the first donor compartment, Hin, Hout, Dt, Db, and H values were kept practically unchanged (Table 1). Therefore, these versions allowed for exploring the role of angulation and the ratios between inlet and outlet diameters, demonstrating that these are critical factors in preventing fluid damming.

Considering versions 3 and 4, we sought to evaluate the role of widening the donor compartment’s lower part to optimize the eyeball coupling in the system. As shown in Table 1, it can be observed that despite the lower diameter Db being the same between the two versions, the parameter Hb (height from where the widening starts) assumes the values of 9.30 mm and 7.15 mm in versions 3 and 4, respectively. The eyeball accommodation tests indicated better performance for the highest Hb value (version 3). In version 5, all the optimization variables explored are finally combined, such as the angle θ, the inlet and outlet diameters, and the inferior enlargement of the donor medium, which has been increased to Hb 12.72 mm. A final variable not explored based on the hydrodynamic flow of the dammed fluid was tested in this model. For this purpose, version 5 was produced to establish a more significant gap between the inlet and outlet heights of the tear flow. 

As expected, the hydrodynamic flow ensured that such optimization in the donor compartment avoided fluid damming. Therefore, considering the results, version 5 was chosen as a donor compartment in the dynamic model.

### 3.2. Mathematical Description of System Clearance Effects

The clearance is a crucial factor for model performance. Here, the clearance percentage was extrapolated from the physiological one. At physiological conditions, the preocular tear film is subjected to a continuous production cycle, evaporation, absorption, and drainage, which leads to a dynamic equilibrium in the preocular tear film. The human basal tear flow in healthy subjects is approximately 1.1 µL/min [36]. Considering a 7–10 µL basal tear volume, this would represent a tear turnover of approximately 11–16%/min [37]. However, a process of reflex flow is initiated in stress conditions as the instillation of medicines, lasting until equilibrium conditions are re-established. Another extrapolation of the present model is the total volume of formulation initially in contact with the eye. In a physiological condition, the ocular globe would hold only approximately 50 µL of formulation on its surface, homogeneously distributed in the eye surface by the eyelids. As the ex vivo model lacks an eyelid, the volume needed to be more prominent because of the tissue curvature to cover the whole cornea surface.

Moreover, the risk of using low volumes in the donor compartment is not obtaining detectable drug amounts in the ocular tissues after 13 min. Therefore, we initially chose to use 300 µL of formulation, ensuring complete coverage of the cornea surface. The rationale was then to submit this applied formulation to a depuration rate that would simulate an initial “reflex response” (for a short period of 2 min), followed by the basal tear turnover (until experiment completion). For such a “reflex response”, we used 16% depuration per minute, followed by 11% depuration, simulating the minimal basal tear turnover. Such a depuration is achieved by operating the peristaltic pump at flows of 48 and 33 µL/min, respectively, considering the use of 300 µL of each formulation.

A mathematical description of formulation depuration effects was calculated, resulting in a theoretical exponentially decreasing profile associated with the formulation volume clearance depending on the flow, described by Equation (1):

(1) $$V_{\left(t\right)}=V_{0}e^{-\beta t},$$

where *V*_(*t*)_, *V*_0_, and *β* represent the volume available after a time interval (*t*) has passed off the depuration, the initial volume of the formulation, and a coefficient related to the speed of clearance of the initial volume (whose unit is defined as the reciprocal of the unit of time, s^−1^), respectively.

**Figure 4:**
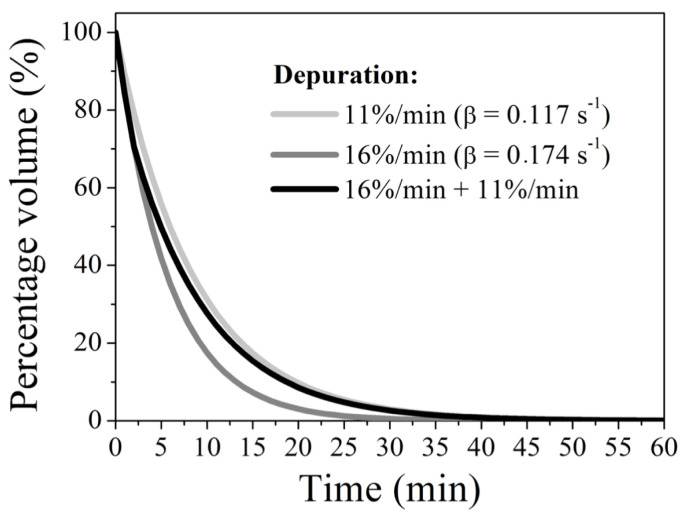
Mathematical simulation of the temporal evolution of the volume percentage available in the donor medium as a function of the used purification. A tear flow of 33 µL/min (11% of depuration/min), 48 µL/min (16% of depuration/min), and a combination of both 48 µL/min (16% of depuration/min) and 33 µL/min (11% of depuration/min) are represented in grey, dark grey and black, respectively.

Assuming that the clearances adopted here (11%/min, 16%/min) are constant and lead to a progressive and non-linear reduction in the amount of formulation in the donor compartment, it was possible to estimate the temporal evolution of the volume available in this compartment. Initially taking as a case study the lowest clearance of 11%/min, it appears that after the first three minutes of testing, the available volume was 89%, 79.21%, and 70.51% of its initial value, respectively. This behaviour reveals an exponentially decreasing profile associated with diluting the formulation volume, demonstrated by Equation (1). Without loss of generality, this mathematical approach was also applied to the case of diluting adjusted to 16%/min. Figure 4 presents the evolution of the volume percentage available in the donor medium as a function of time for clearances of 11%/min and 16%/min, respectively, yielding clearance coefficients of 0.117 s^−1^ and 0.174 s^−1^ for these conditions. As expected, for fixed times, the available volume is greater the smaller the clearance coefficient. For example, comparing both cases, after 15 min of system operation, the volume is reduced to 17.29% and 7.35% of its initial value under diluting of 11 and 16%/min, respectively. A central aspect of this system’s clearance mathematical modelling is the possibility of correlating the available formulation volume with the results obtained in the permeation tests.

Concerning the third condition, the adoption of two flow regimes (cleaning) can be modelled sequentially by estimating the percentage change in two stages: the first stage is expressed as Equation (2) equivalent at the first two minutes, and the second stage is expressed by Equation (3) at the later times. It is important to note that in Equation (2), the *β* coefficient is equivalent to the value found for the highest clearance (16%/min). This is because the initial volume corresponds to the total volume in the donor compartment (300 µL). On the other hand, in Equation (3), the amount available after this first purification regime (occurring in the first two minutes) is assumed as the initial volume, i.e., 211.83 µL. Furthermore, it is essential to note that the clearance coefficient in this second expression corresponds to that found for 11%/min clearance. The subtle change in the temporal parameter led to the change from *t* to *t* − 2, which was necessary because, in this second stage, the first two minutes had already elapsed. Through this sequential description, it is shown that operating the system at 16%/min in the first 2 min and continuing the operation for another 13 min, the percentage value of available volume results in 15.43%, differing from what is found at the same time for the other conditions. In this case, the evolution of the available volume percentage as a function of time follows the bi-exponential profile shown by the black line in Figure 4.

(2) $$V_{\left(t\right)}=300e^{-0.174t}$$

(3) $$V_{\left(t\right)}=211.83e^{-0.117\left(t-2\right)}$$

### 3.3. Operating Conditions of the Dynamic Model

**Figure 5:**
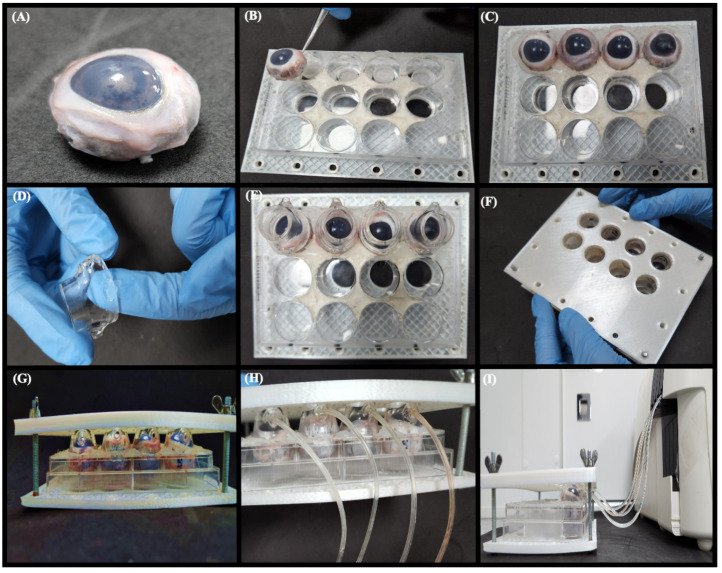
A dynamic model with the lacrimal flow. (**A**) Removal of the eyelids from the eyeball. (**B**) Position the eyeball in a 12-well plate next to the lower 3D support. (**C**) Quadruplicate of the eyeball positioned on the plate, (**D**) Silicone grease is applied to the sides of the donor medium to avoid leakage of the formulation, (**E**) Donor media fixed to the eyeballs. (**F**) Closing the system with the 3D-printed top cover. (**G**) Finished model assembly to couple the flow tubes, flow tubes coupled to the inlet of the donor medium. (**H**) Fully assembled dynamic model. (**I**) System set for the start of the tests.

The dynamic model is assembled in five steps: (I) cleaning all porcine eye globes and removing all the fat, nerves, and eyelids [24]; (II) fitting the eye globes in a 12-well plate; (III) coupling the donor compartment on the eyeball (could be used silicon grease to avoid leaking); (IV) closing the model with 3D-printed plates and screws; and (V) coupling the peristaltic pump tubing in the donor compartment inlet set to 48 µL/min (16% of depuration/min) for 2 min and 33 µL/min (11% of depuration/min) for 13 min (Figure 5 and Supplementary Material).

The operating conditions were established according to the mathematical simulation of the temporal evolution of the percentage of volume available in the donor medium as a function of the used depuration described in Figure 4, seeking to find a relationship between the formulation’s penetration time and the model’s performance.

### 3.4. Ex Vivo Ocular Drug Penetration with the Simulated Lacrimal Flow and Static Model

A permeation experiment challenging the proposed model was performed by comparing FLU permeation from the same formulation sets in a classical Franz diffusion-type static model. Such a challenge employed formulations previously tested in vivo that indeed showed differences in ocular residence time [15] and drug release when compared to a drug solution (SOL) [16], demonstrating that the dynamic mechanisms of eye protection affect such formulation’s performances. 

**Figure 6:**
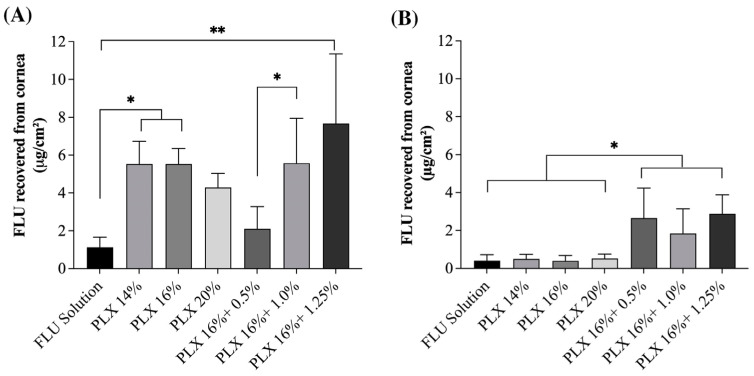
Results of the FLU permeation test in different formulations of PLX and CS. (**A**) Static model: ex vivo corneal FLU penetration results for 15 min. (**B**) Dynamic model: ex vivo corneal FLU penetration results under simulated tear flow (2 min under 16%/min clearance + 13 min under 11%/min clearance). Statistical differences have been verified by the one-way ANOVA test with multiple comparisons. * *p* < 0.05; ** *p* < 0.01.

Figure 6A shows drug penetration data referring to static studies, where the formulation remains in contact with the cornea the entire time of the experiment. In such a condition, the release profile of the drug from the formulation may play a more significant role than in a dynamic condition in which the contact time with the biological membrane is limited. This should explain why there is a trend of higher FLU penetration from the formulations with lower viscosity when there is not a permeation enhancer, i.e., from formulation PLX 14% compared to formulation PLX 20% in static condition. Conversely, in the static condition when a penetration enhancer is added to the formulation, there is a trend of higher FLU permeation with a higher chitosan concentration, demonstrating the permeation enhancer contact with the cornea alters the barrier permeability. Again, such a trend is not observed in a dynamic condition, which might also be explained by the lower contact time of the formulations components with the tissue. Even tough such a trend is not present in the dynamic model, the model was capable of differentiating sets of formulations with different performances. According to Figure 6B, in a dynamic model, it is possible to verify that FLU permeated amounts were independent of the percentage of poloxamer used in the gels (PLX14, PLX16, and PLX20), meaning the viscosity of the formulation had a minor impact compared to the presence of a musoadhesive component [38]. Previous animal and human studies of our group have already confirmed the penetration enhancer capacity of chitosan and the prolonged residence time it confers to the formulation [15,16]. There is no statistical difference (*p* < 0.05) between the different chitosan proportions in the investigated gels in the dynamic model, but, as expected, they showed an increase in FLU permeation compared to poloxamer formulations. This set of results demonstrates that the dynamic model can differentiate the residence time of different formulations and the role of mucoadhesiveness in the tests without the need of animal experimentation. 

Comparing the samples in the static penetration test with a Franz diffusion cell (Figure 6A) and the dynamic model test, the capacity of the dynamic model to differentiate formulations could be confirmed, i.e., formulations with and without chitosan provided similar drug amounts in the cornea following the static penetration experiments, but, when the dynamic model was used, formulations with a mucoadhesive excipient provided significant higher drug amounts in the tissue. Also, the static assays overestimated the permeation values of actives by more than five times in the poloxamer gels and about twice in the case of the gels formulated with chitosan.

It is important to note that when comparing the gels, the static model once again proves that the viscosity does affect the permeation of FLU since the same values are observed for different percentages of poloxamer gels. On the other hand, in poloxamer/chitosan gels, the expected behaviour was observed, in which the greater the chitosan percentage, the greater the mucoadhesiveness and the greater the amount of FLU permeated.

## 4. Discussion

The adaptation of static drug permeation tests by adding a simulated tear flow provided a greater complexity to the test, allowing the establishment of a more sensitive model capable of assessing the impact of the formulation parameters on the drug permeation profile more realistically.

As previously discussed, the formulation clearance cannot be extrapolated considering only the basal tear renewal since, after ophthalmic administration, the reflex defence mechanism begins, culminating in the extra production of tear fluid and mechanical reflexes such as changing the frequency of blinking and drainage increase. The presented model made several extrapolations to simulate such conditions. However, drug drainage from the donor chamber proposed here is not intended to correlate with in vivo bioavailability directly. Still, an ex vivo permeation experiment that considers at least the expected basal clearance can provide information about the expected in vivo formulation performance and help compare sets of formulations.

Undoubtedly, the dynamic model has demonstrated promising results for evaluating ophthalmic drug products and represents a significant step forward regarding reducing and replacing live experimentation animals. Further optimizations should focus on searching for means to replace the animal-extracted eye globe. Indeed, acquiring intact porcine eyes for experimentation can be challenging, as it requires a reliable and timely supply chain. Moreover, to ensure tissue barrier integrity, experiments must be performed on the same day of the extraction, which may pose additional logistical constraints in experimental design and implementation. The lack of dynamic components, i.e., the absence of blinking mechanics, which play a crucial role in ocular physiology and the distribution of tear film, may limit the model’s ability to fully capture the complex interactions between the eye and tear flow. Hence, future advancements addressing such limitations may be expected shortly. 

## 5. Conclusions

The dynamic model with simulated tear flow is a promising new alternative model for the performance evaluation of ophthalmic drug products and represents a step forward regarding the reduction and replacement of live experimentation animals. Parameters of assembly, optimization, and operation of the dynamic model have been successfully established, enabling its complete application during the research and development of ophthalmic formulations. Furthermore, the results demonstrate that the model can differentiate innovative formulations, therefore representing a more realistic alternative to classical static experiments.

## Figures and Tables

**Table 1:** Geometric parameters evaluated in the donor compartment tested versions.

N°	Donor Compartment Parameters (mm)							Geometric Parameters (mm)			
	d_in_	d_out_	d_out_/d_in_	H_in_	H_out_	H_in_/H_out_	θ	D_t_	D_b_	H_b_	H
1	2.05	3.61	1.76	7.44	8.70	0.85	90°	12.18	-	-	43.96
2	2.61	5.34	2.04	4.30	3.00	1.43	<90°	14.27	-	-	30.17
3	1.29	2.9	2.24	13.0	13.0	1.00	<90°	14.37	20	9.30	28.08
4	1.26	2.95	2.34	10.8	11.5	0.93	90°	14.37	20	7.15	20.14
5	2.43	5.00	2.05	10.1	5.60	1.80	<90°	13.38	20	12.72	18.63

## Data Availability

The data presented in this study are available in this article and supplementary material.

## Supplementary Materials

The following supporting information can be downloaded at: https://www.mdpi.com/article/10.3390/pharmaceutics15092325/s1. Protocol assembly of the Dynamic ex vivo Porcine Eye Model.
