# Supplementary material for: Dynamic Ex Vivo Porcine Eye Model to Measure Ophthalmic Drug Penetration under Simulated Lacrimal Flow
Source: Pharmaceutics. 2023 Sep 15;15(9):2325. doi: 10.3390/pharmaceutics15092325 (PMC10534681; doi:10.3390/pharmaceutics15092325)

Protocol assembly of the Dynamic *ex vivo* Porcine Eye Model

Step 1: First the whole eye globe needs the ocular dissection. A) Eyeball with eyelids. B) Removal of the eyelids from the eyeball. C) Clean eyeball, ready for the experiment.

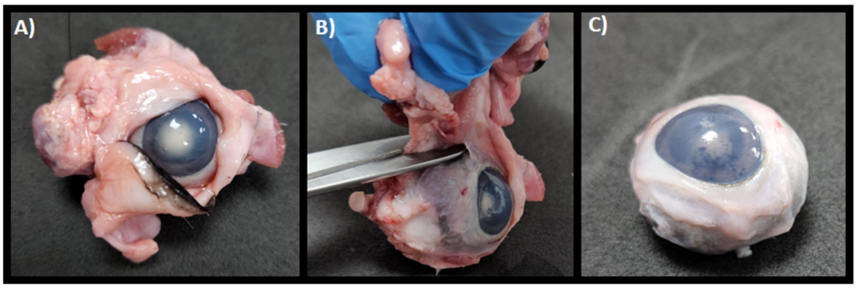

Step 2: Assembling the eyeball fitting base. D) 12-well plate positioned next to the lower 3D support. E) and F) Positioning of the eyeball. G) Quadruplicate of eyeball positioned on the plate.

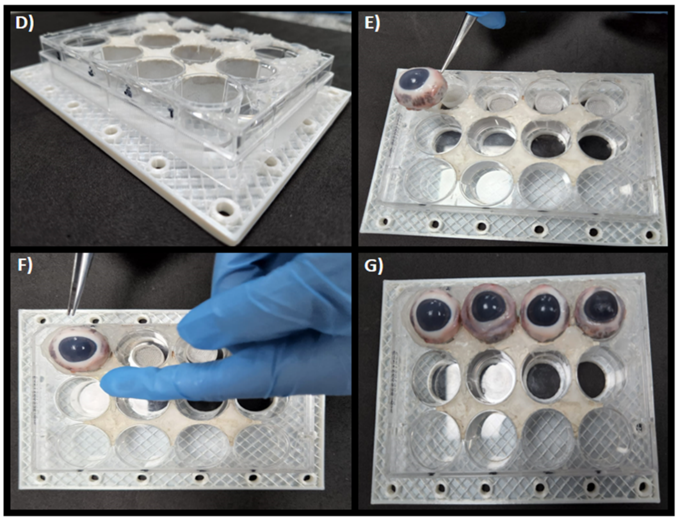

Step 3: Top assembly of the Dynamic *ex vivo* porcine eye model. H) Silicone grease is passed on the sides of the donor medium to prevent formulation leakage. I) Donor media fixed to the eyeballs. J) System closure with 3D printed top cover. K) Completed model assembly to attach flow tubes.

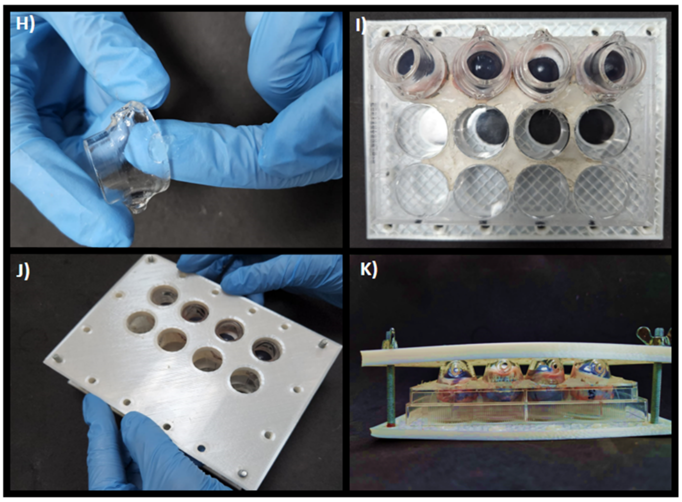

Step 4: Model showing exposed corneas. M) Flow tubes attached to the inlet of the donor medium. N) Fully assembled Dynamic model. On the left, the fixed donor and receptor media, in the center the peristaltic pump and on the right a beaker containing the lacrimal solution.

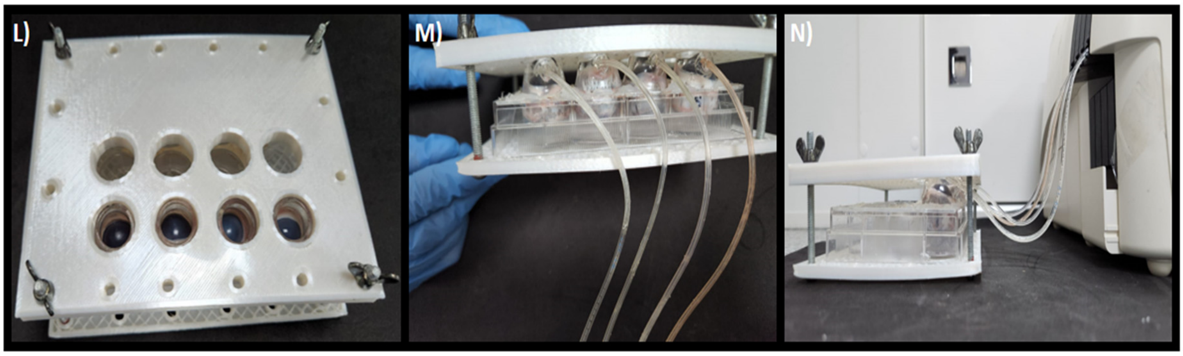

Step 5: Add 300 μL of formulation in the top of the eye and adjust the peristaltic pump for 2 minutes of 16%/min clearance + 13 minutes under 11%/min clearance) and started the penetration experiment.

Step 6: O) Removal of the eyeball after the test. P) Washing the entire eyeball with ultra-pure water to remove excess formulation. Q) Perforation of the eyeball with a scalpel. A) Removal of the cornea with the scissors. S) Cornea washing with ultra-pure water after removal. T) Excised cornea ready for the drug extraction process.

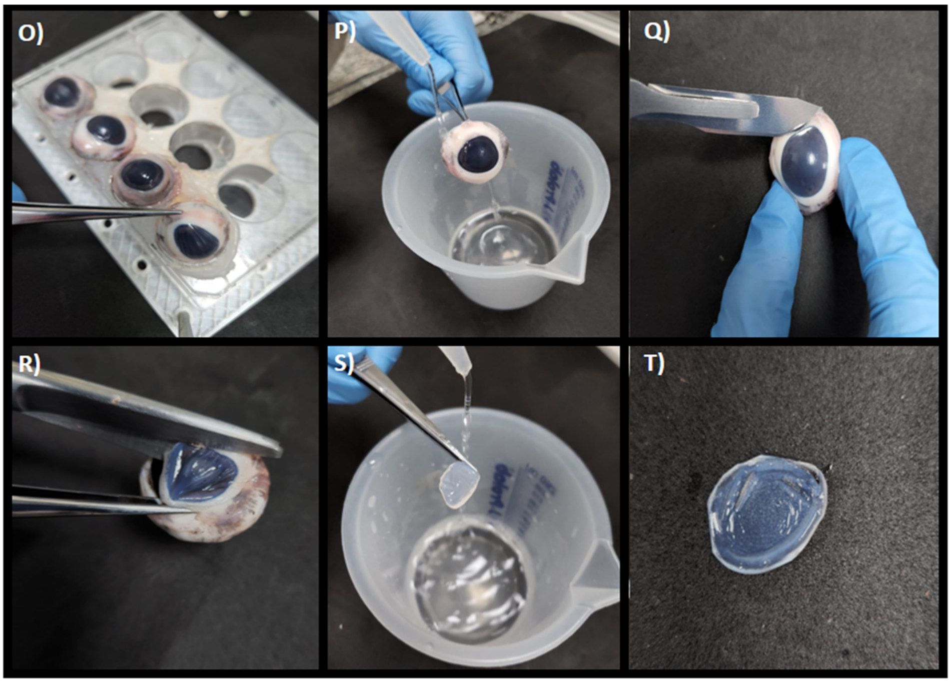

Step 7: For the drug extraction process, you need to choose the method according to the drug and the method analyses, for example: Confocal images, HPLC, fluorescence microscopy and others.

Supplementary data

For 3D printed bases, ABS (Acrylonitrile Butadiene Styrene) filament was used following these parameters. For the fixed model was used metal screw and a screw nut.

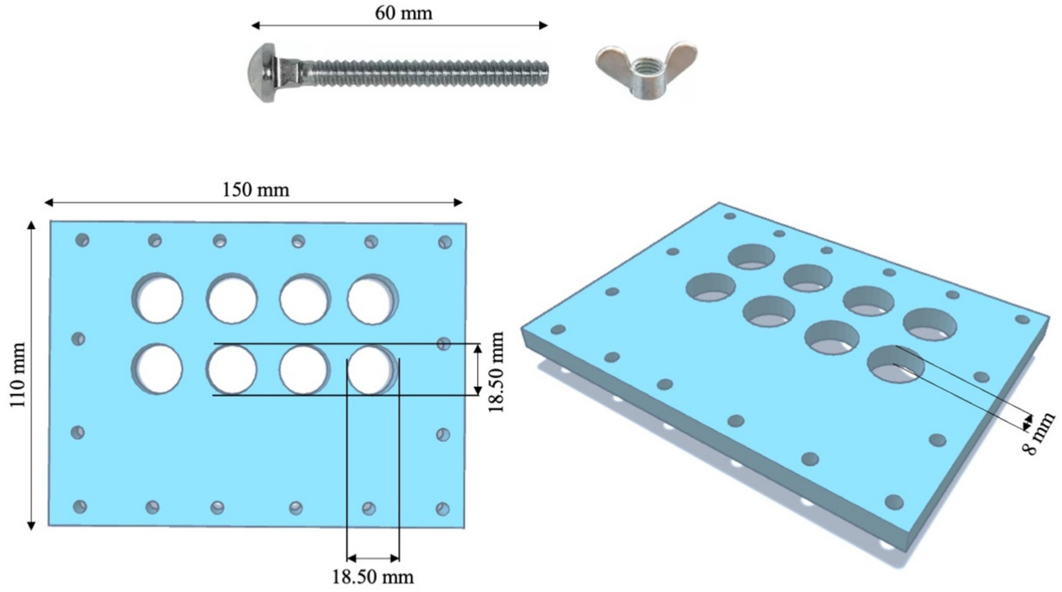

Supplement: Supplementary file 1 [file pharmaceutics-15-02325-s001.zip › pharmaceutics-2592871-supplementary.pdf]
